# Targeting EZH2-mediated methylation of H3K27 inhibits proliferation and migration of Synovial Sarcoma *in vitro*

**DOI:** 10.1038/srep25239

**Published:** 2016-04-29

**Authors:** Jacson K. Shen, Gregory M. Cote, Yan Gao, Edwin Choy, Henry J. Mankin, Francis J. Hornicek, Zhenfeng Duan

**Affiliations:** 1 Sarcoma Biology Laboratory, Center for Sarcoma and Connective Tissue Oncology, Massachusetts General Hospital, Boston, United States; 2 Division of Hematology and Oncology, Massachusetts General Hospital, Boston, United States

## Abstract

Synovial sarcoma is an aggressive soft tissue sarcoma genetically defined by the fusion oncogene SS18-SSX. It is hypothesized that either SS18-SSX disrupts SWI/SNF complex inhibition of the polycomb complex 2 (PRC2) methyltransferase Enhancer of Zeste Homologue 2 (EZH2), or that SS18-SSX is able to directly recruit PRC2 to aberrantly silence target genes. This is of potential therapeutic value as several EZH2 small molecule inhibitors are entering early phase clinical trials. In this study, we first confirmed EZH2 expression in the 76% of human synovial sarcoma samples. We subsequently investigated EZH2 as a therapeutic target in synovial sarcoma *in vitro*. Knockdown of EZH2 by shRNA or siRNA resulted in inhibition of cell growth and migration across a series of synovial sarcoma cell lines. The EZH2 selective small-molecule inhibitor EPZ005687 similarly suppressed cell proliferation and migration. These data support the hypothesis that targeting EZH2 may be a promising therapeutic strategy in the treatment of synovial sarcoma; clinical trials are initiating enrollment currently.

Aberrant epigenetic changes are increasingly being recognized as important drivers of malignancy, including altered covalent histone amino tail modifications (e.g. acetylation, methylation, etc.), hyper- or hypo-DNA methylation, and dysregulation of the ATP-dependent chromatin remodeling enzymes that regulate these processes. ATP-dependent chromatin remodeling occurs via several intricate multiprotein complexes, which are thought to alter the nucleosome structure, thus regulating chromatin packaging and transcription (for a comprehensive review see Wilson *et al.*^1^ and Mermoud *et al.*^2^). There are five known ATP-dependent chromatin complexes of which the best characterized is SWI/SNF ([SWItch/Sucrose Non-Fermenting] several variants exist). SWI/SNF may function to either slide or remove the histone from the nucleosome and thereby alter the degree of chromatin compaction (i.e. histone ejection from the nucleosome would open the chromatin and thus allow access of RNA polymerase complexes)^3^,^4^. In contrast, the polycomb group repressive complexes (PRC1 and PRC2) work in concert to modify histone tails to suppress transcription^4^,^5^. The PRC2 component Enhancer of Zeste Homolog 2 (EZH2) is a potent histone methyltransferase that targets histone 3 lysine 27 (H3K27)^6^,^7^. Current models suggest antagonistic functions of SWI/SNF (activating transcription) and PRC2/EZH2 (silencing transcription) to fine tune levels of gene expression^6^,^8^,^9^.

Emerging data support SWI/SNF as an epigenetic tumor suppressor and EZH2 as an oncogene. Human tumor sequencing studies have identified multiple alterations in SWI/SNF across a range of malignancies^10^ and activating EZH2 mutations in lymphoma (and over-expression in other tumors)^7^,^11^,^12^,^13^,^14^, and in some these have been correlated with worse clinical outcomes^15^. Some examples of the SWI/SNF-PRC2/EZH2 oncogenesis hypothesis include malignant rhabdoid tumors (MRT) in which the SWI/SNF subunit INI1 (a.k.a. hSNF5, SMARCB1, BAF47) is inactivated, and in lymphomas with EZH2 activating mutations. In preclinical models of MRT, rescue of INI1 or inhibition of EZH2, and lymphoma (with EZH2 activating mutations), inhibition of EZH2 leads to slowing of tumor growth and apoptosis^16^,^17^,^18^,^19^,^20^,^21^. Moreover, these data are now supported by early phase clinical studies where EZH2 inhibitors have shown clinical responses in MRT and lymphoma (unpublished data). Could this strategy be relevant to other sarcomas with INI1/EZH2 alteration? INI1 mutation or loss of expression has been described in connective tissue malignancies, including round cell soft-tissue sarcomas (most were a subset of tumors resembling extraskeletal myxoid chondrosarcoma with rhabdoid features)^22^, epithelioid sarcomas^23^, and in poorly differentiated chordoma^24^. These are all extremely rare histologies (e.g. we estimate there are less than 10 cases of poorly differentiated chordoma diagnosed in the US per year) and as such, clinical study remains difficult. Synovial sarcoma is the third-most common soft tissue malignancy in adolescents and young adults, and accounts for up to 10% of all soft tissue sarcomas^25^. Standard treatment involves multimodality therapy including surgery with or without radiation and/or chemotherapy^26^,^27^. However, despite this aggressive approach relapse is not uncommon and in the metastatic setting synovial sarcoma is universally fatal^28^,^29^. Molecularly, synovial sarcoma is defined by the chromosomal translocation t(X;18)(p11.2;q11.2), which fuses the SS18 (SYT, a SWI/SNF subunit^30^,^31^) gene to either SSX1, SSX2, or rarely SSX4^28^,^32^. The aberrant fusion protein SS18-SSX fusion is believed to derive its oncogenic activity through altered transcription^33^. Recent models suggest two potential competing mechanisms of transforming activity in synovial sarcoma. SS18-SSX displaces wild-type SS18 and INI1 from the SWI/SNF complex and this may drive Sox2-mediated proliferation/dedifferentiation^31^. Alternatively, there is evidence that SS18-SSX may directly recruit PRC2 and HDAC to ATF2 targets and thus silence transcription at those loci^34^. Previous studies have implicated the role of the SS18-SSX fusion genes in epigenetic regulation and modification of target genes^35^,^36^,^37^,^38^.

In summary evidence suggests that imbalance of SWI/SNF-PRC2/EZH2 can drive oncogenesis in sarcomas. In synovial sarcoma the SS18-SSX may drive aberrant gene silencing through either loss of EZH2 inhibition or by direct recruitment to polycomb targets. We and others hypothesize that inhibition of EZH2 is a potential clinical therapeutic target in synovial sarcoma^39^,^40^,^41^. This study demonstrates that synovial sarcoma is dependent on EZH2 for tumor survival and migration.

## Results

### EZH2 expression in synovial sarcoma human tissue

We sought to confirm that high-level EZH2 expression is present in human synovial sarcoma samples. The tissue samples were taken from 20 female and 30 male patients with a median age of 40 years. Semi-quantitative EZH2 expression staining patterns were categorized into 6 groups: 0, no nuclear staining; A, 1+, <10% of cells stained positive; B, 2+, 10% to 25% positive cells; C, 3+, 26% to 50% positive cells; D, 4+, 51% to 75% positive cells; and E, 5+, >75% positive cells. Tumor cell staining was nuclear as anticipated. Of the 50 cases, 76% displayed detectable EZH2 expression; the staining intensities were as follows: 1+ = 22%, 2+ = 22%, 3+ = 14%, 4+ = 12% and 5+ = 6% (Supplementary Figure S1A). We evaluated the clinical history of the human tumor specimens and found no significant correlation between EZH2 expression and tumor tissue type/location, TNM stage, and pathology grade. There were, however, differences in EZH2 expression between clinical stages; 13 patient specimens were in stage I, 16 in stage II, 20 in stage III, and one in stage IV. Patients with stage II and stage III synovial sarcoma had significantly higher EZH2 scores (2.188 ± 0.3788, p = 0.0437 and 2.200 ± 0.3524, p = 0.0388, respectively) than stage I patients (1.077 ± 0.3483). The individual patient in stage IV did not show any EZH2 staining expression (Supplementary Figure S1B).

### Knockdown of EZH2 inhibits synovial sarcoma cell line proliferation

The Aska-SS and Yamato-SS cell lines were confirmed to contain the SS18-SSX1 fusion gene variant and the Fuji and SYO-1 cell lines were shown to have the SS18-SSX2 fusion gene variant by quantitative RT-PCR (Supplementary Figure S2).

**Figure 1:**
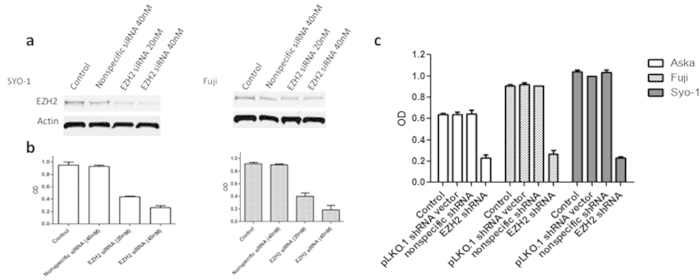
Knockdown of EZH2 by synthetic siRNA or lentiviral shRNA inhibits proliferation in synovial sarcoma cell lines. (**a**), Confirmation of EZH2 protein expression knockdown after siRNA transfection in SYO-1 and Fuji cell lines by Western blot. (**b**), Inhibition of cell proliferation after EZH2 siRNA transfection in SYO-1 and Fuji cell lines as determined by the MTT assay. (**c**), Inhibition of cell proliferation after EZH2 shRNA transduction in Aska-SS, Fuji, and SYO-1 cell lines as determined by MTT assay. Western blot images were cropped to improve the conciseness of the data; samples derive from the same experiment and the blots were processed in parallel.

The Fuji and SYO-1 cell lines were transfected with human EZH2 siRNA. After 96 hour cultures siRNA treatment resulted in a knockdown of EZH2 in both cell lines (Fig. 1A), as well as dose-dependent inhibition of proliferation (Fig. 1B). To confirm these findings, lentiviral human EZH2 shRNA was transduced into the Aska-SS, Fuji, and SYO-1 synovial sarcoma cell lines. After establishing subcultured stable cell lines through puromycin selection, proliferation was assessed by the MTT assay after 96 hours. Knockdown of EZH2 by shRNA inhibited proliferation in all cell lines (Aska-SS, Fuji, and SYO-1) while the growth in the control cell lines was unaltered (Fig. 1C) similar to siRNA above.

### Knockdown of EZH2 suppresses synovial sarcoma cell migration *in vitro*

EZH2 is the only known methyltransferase of H3K27me3;^42^ thus, to confirm on-target effect, the knockdown of EZH2 expression and its trimethylation function were analyzed after 72 hours of treatment. *In vitro* cell line migration was assessed by a semi-quantitative wound-healing recovery assay. EZH2 siRNA was transfected into Yamato-SS, Aska-SS, and SYO-1 synovial sarcoma cell lines as described. Following 24 hours of treatment and 48 hours after the wounding all cell lines demonstrated inhibition of wound healing with EZH2 siRNA transfection compared with control lines.

**Figure 2:**
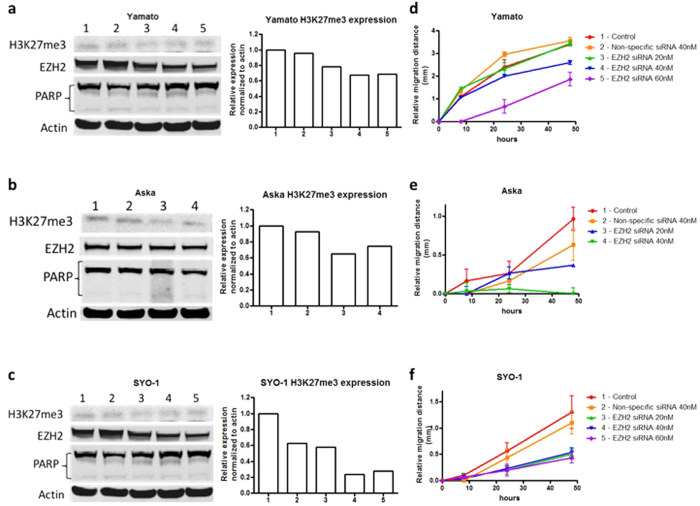
Knockdown of EZH2 by synthetic siRNA restricts migration in synovial sarcoma cell lines *in vitro*. Confirmation of knockdown of EZH2 protein and H3K27me3 expression after EZH2 siRNA transfection in Yamato-SS (**a**), Aska-SS (**b**), and SYO-1 (**c**) cell lines as determined by Western blot and densitometry. No significant difference in PARP cleavage between the different concentrations indicated that the inhibition of migration was not related to cell viability. Suppression of cell migration distance after EZH2 siRNA transfection in Yamato-SS (**d**), Aska-SS (**e**), and SYO-1 (**f**) cell lines as determined by the wound healing assay. Western blot images were cropped to improve the conciseness of the data; samples derive from the same experiment and the blots were processed in parallel.

Western blot results demonstrated a concentration-dependent inhibition of EZH2 expression, and densitometric analysis showed decreasing H3K27me3 expression in each of the cell lines, Yamato-SS, SYO-1, and Aska-SS (Fig. 2A–C). To rule out apoptotic cell death as the mechanism of suppressed migration, poly(ADP-ribose) polymerase (PARP) cleavage was assessed. There was no significant difference in PARP cleavage between the different concentrations in any cell line at 72 hours, indicating that the inhibition of migration was not related to cell viability (Fig. 2A–C).

The Yamato-SS cell line was nearly completed recovered after 48 hours of migration in the control and non-specific siRNA transfected cells, covering 3.4 ± 0.027 mm and 3.6 ± 0.15 mm, respectively (Fig. 2D). EZH2 siRNA-treated cell line showed inhibition of wound healing at 40 nM (2.6 ± 0.093 mm) and 60 nM (1.8 ± 0.31 mm) only with no effect at 20 nM (3.4 ± 0.11 mm).

EZH2 siRNA transfection also suppressed migration in the Aska cell line. EZH2 siRNA transfected cells migrated 0.97 ± 0.15 mm and 0.63 ± 0.21 mm, respectively, whereas cells transfected with 20 nM of EZH2 siRNA migrated 0.37 ± 0.00 mm, and greater concentrations of siRNA resulted in no migration and cell detachment (Fig. 2E).

**Figure 3:**
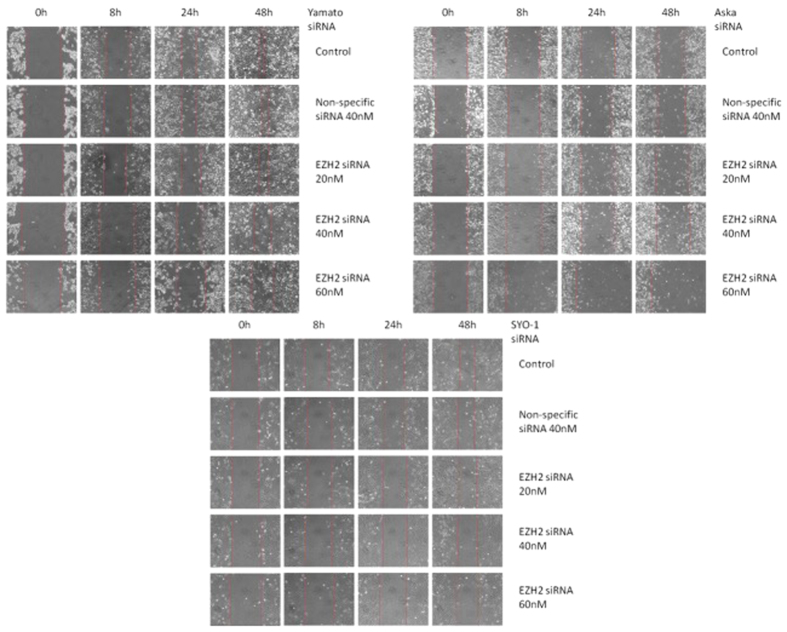
Migration images of EZH2 siRNA transfected synovial sarcoma cell lines. Yamato-SS, Aska-SS, and SYO-1 cell lines were treated with the specified siRNA concentrations at 0, 8, 24, and 48h time points.

Similar migration results were also demonstrated in SYO-1 cells, which travelled 0.50 ± 0.095 mm, 0.53 ± 0.087 mm, and 0.43 ± 0.10 mm with 20 nM, 40 nM, and 60 nM, respectively, while control and non-specific siRNA cells covered 1.3 ± 0.31 mm and 1.1 ± 0.21 mm, respectively (Fig. 2F). Cell migration images are shown in Fig. 3.

### EPZ005687 specifically targets EZH2 methyltransferase function on H3K27me3

**Figure 4:**
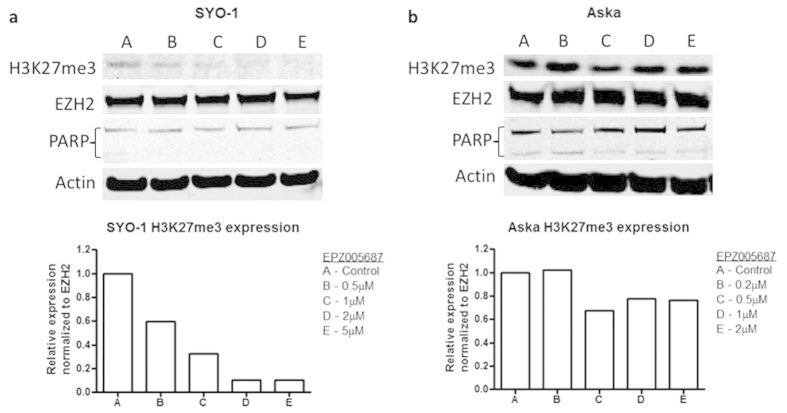
EPZ005687 specifically targets EZH2 methyltransferase function on H3K27me3. H3K27me3 expression was examined in SYO-1 (**a**) and Aska-SS (**b**) cell lines by Western blot and densitometry to confirm target specificity of EPZ005687 on EZH2 function and not EZH2 itself. Additionally, there was no significant difference in PARP cleavage between the different concentrations in the cell lines. Western blot images were cropped to improve the conciseness of the data; samples derive from the same experiment and the blots were processed in parallel.

EPZ005687 is a specific inhibitor of EZH2^16^, and as such H3K27 trimethylation status is a direct marker of PRC2 activity. Therefore, H3K27me3 protein expression was assessed in synovial sarcoma cell lines after treatment with EPZ005687. Aska-SS and SYO-1 cells were treated with increasing concentrations of EPZ005687 and were then assessed for H3K27me3 status after 72 hours, apoptosis after 72 hours, proliferation after 14 days and migration over 48 hours with 72 hours of constitutive treatment. Western blot and densitometry results demonstrated a dose-dependent inhibition of H3K27me3 with EPZ005687 (Fig. 4A,B). The expression of level of EZH2 remained constant between treated and indicating inhibition of enzymatic activity without degradation of EZH2 protein.

### EPZ005687 inhibits synovial sarcoma cell proliferation and migration

**Figure 5:**
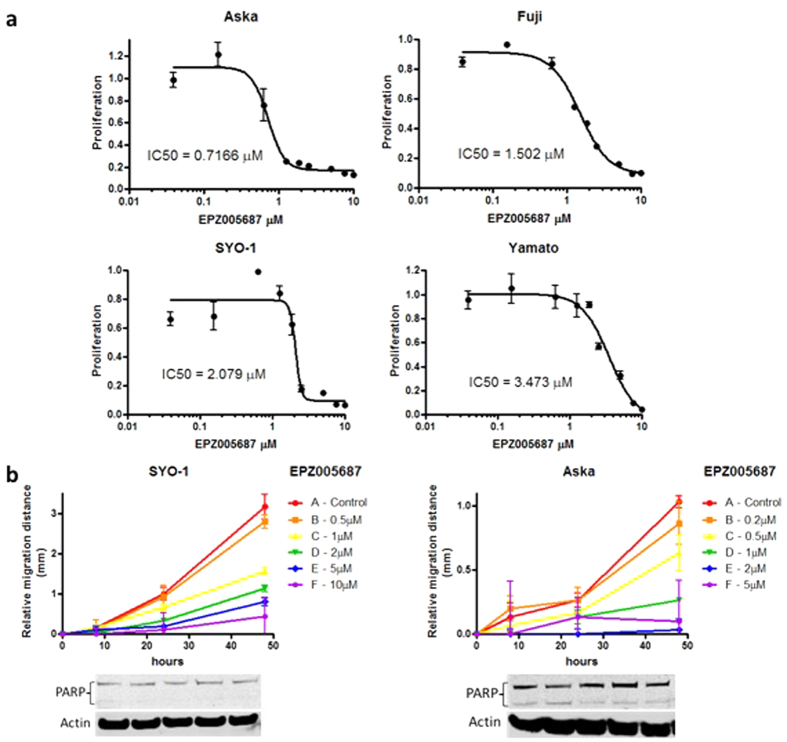
EPZ005687 inhibits synovial sarcoma cell proliferation and migration. (**a**), The IC50 of Aska-SS, Fuji, SYO-1, and Yamato-SS treated with EPZ005687 over 14 days as evaluated by the MTT assay. (**b**), Migration distances of SYO-1 and Aska-SS cell lines after 72 h treatment with EPZ005687 as evaluated by the wound healing assay. Western blot images were cropped to improve the conciseness of the data; samples derive from the same experiment and the blots were processed in parallel.

To examine the effect of EPZ005687 on synovial sarcoma cell proliferation, Aska-SS, Yamato-SS, Fuji, and SYO-1 cells were treated with increasing concentrations of EPZ005687 over 14 days. Inhibition of cell proliferation by EPZ005687 in each cell line was determined by the MTT assay. Over 14 days, the non-viable cells appeared to detach from the plate. The IC50 of EPZ005687 in Aska-SS was 0.72 μM, Fuji was 1.5 μM, SYO-1 was 2.1 μM, and Yamato-SS was 3.5 μM (Fig. 5A). The chordoma UCH2 cell line and the liposarcoma SW872 cell line do not contain the SS18-SSX translocation, and thus, were used as controls. Both cell lines demonstrated limited inhibition of proliferation (Supplementary Figure S3).

We further examined the effect of EPZ005687 on synovial sarcoma cell migration over 48 hours with 72 hours of constitutive treatment. Concentration gradient ranges were adjusted according to the IC50 determined above. Both SYO-1 and Aska-SS cell migration was inhibited with EPZ005687 treatment in a dose-dependent manner with an observable threshold below their IC50s (<1 μM and <0.5 μM, respectively), as compared with untreated control cells (Fig. 5B; Supplementary Figure S4). PARP cleavage showed no significant difference between drug concentrations, indicating that suppression of migration was not related to cell viability (Fig. 5B).

### EPZ005687 in combination with chemotherapy in synovial sarcoma cell lines

To examine whether inhibition of EZH2 could sensitize synovial sarcoma cells to chemotherapeutic drugs *in vitro*, the Aska-SS and SYO-1 cells lines were treated with increasing concentrations of EPZ005687 and etoposide, topotecan, or doxorubicin over 14 days, and IC50s were determined by the MTT assay.

There was no significant synergy of EPZ005687 with the chemotherapy drugs in each cell line. In the SYO-1 cell line, the IC50 of etoposide alone was 0.037 μM, whereas treatment with EPZ005687 decreased the IC50 to 0.026 μM. For topotecan, the IC50 decreased from 0.00065 μM to 0.00060 μM when combined with EPZ005687. Doxorubicin alone exhibited an IC50 of 0.00089 μM, and 0.00070 μM with EPZ005687. The Aska cell line also had a similar trend with combination treatments. The IC50 of etoposide alone was 0.066 μM, and 0.029 μM in combination with EPZ005687; topotecan alone was 0.0038 μM, and 0.0025 μM with EPZ005687; and doxorubicin alone was 0.011 μM, and 0.0019 μM together with EPZ005687 (Supplementary Figure S5).

## Discussion

Growing evidence has identified supports the SWI/SNF-PRC2/EZH2 axis as a powerful tumor suppressor/oncogene pathway. Alterations in SWI/SNF-PRC2/EZH2 are now recognized in a number of tumors and they may correlate with aggressive clinical features^7^,^10^,^11^,^12^,^13^,^14^,^20^,^43^,^44^,^45^,^46^,^47^,^48^,^49^,^50^. Preclinical work and ongoing early phase clinical trials support targeting EZH2 in INI1 or EZH2 altered malignancies. In synovial sarcoma, where SS18-SSX is aberrantly expressed, it is hypothesized that EZH2 overactivity, either from loss of SWI/SNF-mediated EZH2 inhibition or by direct recruitment of EZH2 to polycomb targets, leads to tumorigenesis.

We first confirmed the presence of EZH2 in human synovial sarcoma samples. There was a higher-level expression in stages 2 and 3 in this limited sample. No expression was seen in the stage 4 sample. Due to this limited sample size, it would be useful to expand these investigations to a larger scale study. We believe this is an inherent limitation of the TMA (e.g. sample bias) as Changchien *et al*. previously was able to show high levels of EZH2 expression in metastatic samples^39^.

We next explored RNA knockdown and pharmacologic inhibition EZH2 in synovial sarcoma *in vitro*. We demonstrate robust target inhibition with the shRNA and siRNA and in long-term culture growth inhibition and cell migration. This appears to be independent of any early apoptotic event as PARP is essentially normal. It is important to note, however, that over the course of the long-term cultures some cells would lift off the plate. The mechanism of cell death was unknown and technically assays to evaluate apoptosis were not feasible. It is possible that these data are consistent with an epigenetic event that requires time to evolve, rather than immediate inhibition of a kinase oncogene. For example, this would be consistent with the work from Naka *et al*. where siRNA of SS18-SSX appeared to induce differentiation rather than tumor cell line death^51^.

EPZ005687 is a high-affinity inhibitor for EZH2. Patient trials of the clinical compound Tazemetostat are ongoing in Europe and planned for the US in 2016 (NCI NCT02601937 and NCT02601950). In the above work, we treated synovial sarcoma cell lines with EPZ005687. We saw a dose-dependent decrease in H3K27me3 in the SYO-1 cell line with variable decrease in the Aska-SS cell line. Of note, despite the limited inhibition on Western blotting of H3K27me3 in Aska-SS, there was correlation with increasing dose and inhibition of proliferation in the dose-response curve (Fig. 5) confirming on-target inhibition. Indeed, all cell lines had inhibited proliferation with EPZ005687. The variable IC50 levels likely reflect a combination of drug-target potency and the kinetics of the cell line. For example, SYO-1 grows much more efficiently as compared to Aska-SS and thus the long-term growth curves are more consistent between experiments. For controls, we used liposarcoma and chordoma cell lines, not known to have alterations in SWI/SNF members or EZH2. These cell lines were less sensitive to the inhibitors (Supplementary Figure S3); however, there was a threshold where both lines displayed some level of growth inhibition. It is possible that at high doses EZH2/polycomb is functional or there is some off-target pathway inhibition.

Our data also demonstrate that EZH2 inhibition by RNA knockdown or pharmacologically inhibits cell migration. Further work will be needed to determine the mechanism of this finding. Interestingly, EZH2 has been correlated with metastatic pathways in other cancers. EZH2 is thought to regulate invasion by suppressing Early Growth Response-1 (EGR1), Ras, NF-κB, and the Raf-1 kinase. EGR1 expression was found to decrease the metastatic potential in a number of cancers, such as non-small cell lung cancer, hepatocarcinoma, and fibrosarcoma^52^,^53^,^54^. EGR1 is repressed by the SS18-SSX fusion protein in synovial sarcoma in that SS18-SSX recruits PcG proteins, including EZH2 and Bmi1, to the EGR1 promoter and correlates with H3K27me3^38^. It has also been demonstrated that EZH2 activates the Ras and NF-κB pathways in prostate cancer^55^. Specifically, the Ras GTPase-activating protein (RasGAP) gene DAB2IP is suppressed by EZH2, which in turn activates the metastatic pathway progression through Ras and NF-κB. Furthermore, in breast and prostate cancer EZH2 downregulates the Raf-1 kinase inhibitor protein (RKIP) potentially leading to tumor progression and metastasis^47^.

Recent work has demonstrated synergism of combining EZH2 inhibition with chemotherapy in NSCLC and melanoma, particularly topoisomerase inhibitors and platinum^56^,^57^. This is attractive as it may expand the activity of these agents in clinic. In our work, however, we were unable to demonstrate a significant synergy of EPZ005687 in combination with topotecan, etoposide and doxorubicin. Other combinations and novel agents may be explored in future work.

In summary, the fusion protein SS18-SSX in synovial sarcoma is believed to promote oncogenesis though either loss of negative regulation of EZH2/polycomb or by direct recruitment of EZH2/polycomb. In this study we show that RNA knockdown or pharmacologic targeting of the EZH2 methyltransferase activity inhibits proliferation and migration across a range of synovial sarcoma cell lines. We believe this work and others provides the foundation to explore clinical trials of EZH2 inhibitors in patients with this disease.

## Materials and Methods

### Cell lines, cell culture, and study agents

The human synovial sarcoma cell lines Aska-SS and Yamato-SS which carry the SS18-SSX1 fusion gene were kindly provided by Dr. Kazuyuki Itoh (Osaka Medical Center for Cancer and Cardiovascular Diseases, Osaka, Japan)^51^. The human synovial sarcoma cell line SYO-1 and Fuji which carry the SS18-SSX2 fusion gene were generously provided by Dr. Akira Kawai (National Cancer Center Hospital, Tokyo, Japan)^58^ and Dr. Kazuo Nagashima (Hokkaido University School of Medicine, Hokkaido, Japan)^59^, respectively. The human chordoma UCH2 cell line was kindly provided by Dr. Silke Bruderlein (University Hospitals of Ulm, Germany)^60^, and the human liposarcoma SW872 cell line was purchased from the American Type Culture Collection (Maryland, USA). The Aska, Yamato, SYO-1, and UCH2 cell lines were incubated in DMEM (Invitrogen, Carlsbad, CA) supplemented with 10% fetal bovine serum, 100 U/mL penicillin, and 100 mg/mL streptomycin (Life Technologies, Carlsbad, CA), and the Fuji and SW872 cell lines in RPMI-1640 (Life Technologies, Grand Island, NY) complete media. All cells were maintained in a humidified incubator containing 5% CO_2_–95% air atmosphere at 37 °C. Light microscope images were obtained by a Zeiss microscope from Carl Zeiss, Inc. (Oberkochen, Germany) with an attached Nikon D40 digital camera (New York, NY).

EPZ005687 (Epizyme Inc., Cambridge, MA) is a specific inhibitor of EZH2 activity^16^. EPZ005687 was dissolved in dimethyl sulfoxide (DMSO) and administered at gradient concentrations to synovial sarcoma cells over 72 hours for wound healing and western blot analysis. To evaluate drug cytotoxicity, cells were treated for a total of 14 days following the manufacturer’s protocol with minor modifications. Briefly, 1.5 × 10^5^ cells were seeded in culture medium in a 12-well plate. Fresh media and drug were exchanged on day 4, and cells were reseeded to 1 × 10^4^ cells per well into 96-well plates in triplicate on day 7 with fresh media and drug. Fresh media and drug were again exchanged on day 11, and cell viability was determined by the MTT assay on day 14.

Etoposide (Toposar™, Teva product #00703565301), topotecan (Hospira product #00409030201), and doxorubicin (APP Pharmaceuticals product #63323088305) were obtained from McKesson Medical-Surgical (Richmond, VA). Synovial sarcoma cells were treated at gradient concentrations over 14 days alone or in combination with EPZ005687 for synergism assays, and proliferation was determined by the MTT assay.

### Quantitative real-time RT-PCR

To confirm which fusion gene variant in each of the cell lines, total RNA was extracted using TRIzol Reagent^®^ (Life Technologies) according to the manufacturer’s protocol. RNA samples were quantified using the ultraviolet spectrophotometer at 260 nm (Beckman DU-640, Beckman Instruments, Fullerton, CA). Equal amounts of RNA were reverse transcribed using a High-Capacity cDNA Reverse Transcription Kit (Applied Biosystems, Foster City, CA). cDNAs were then amplified by real-time PCR using TaqMan^®^ gene expression assays (Applied Biosystems) for SS18-SSX1 (Product ID: Hs03024820_ft; probe sequence: 5′-ccagc agaggacgaa aatgattcga agggagtgtc agaagcatct ggcccacaaa acgatgggaa acaactgcac cccccagg-3′) and SS18-SSX2 (Product ID: Hs03024398_ft; probe sequence: 5′-ccagc agaggaagga aatgattcgg aggaagtgcc agaagcatct ggcccacaaa atgatgggaa agagctgtgc cc-3′) according to the manufacturer’s protocol. PCR was performed with TaqMan^®^ gene expression master mix (Applied Biosystems) using 2 μL of cDNA in a 20-μL final reaction volume. The amplification cycles were performed by Applied Biosystems StepOnePlus™ System (Applied Biosystems) as follows: 50 °C for 2 min, 95 °C for 10 min, followed by 40 cycles of 95 °C for 15 seconds, and finally 60 °C for 1 minute. The housekeeping gene β-actin (Product ID: Hs01060665_g1; probe sequence: 5′-ggc gtgatggtgg gcatgggtca gaaggattcc tatgtgggcg acgaggccca gagcaagaga-3′) expression level was used as an internal control to evaluate the integrity of each sample.

### Synthetic EZH2 siRNA transfection

EZH2 knockdown in human synovial sarcoma cells was performed by siRNA transfection. The human EZH2 siRNA (Sigma-Aldrich, SASI_Hs01_00147882) target sequence was as follows: sense: CAUCGAAAGAGAAAUGGAAdTdT; antisense: UUCCAUUUCUCUUUCGAUGdTdT. The nonspecific siRNA oligonucleotides (Qiaqen, Hilden, Germany) were used as negative controls. Synovial sarcoma cells were either cultured on 12-well plates for wound healing and western blot analysis or 96-well plates for cell proliferation assays. Various concentrations (0, 20, 40, and 60 nM) of EZH2 siRNA or nonspecific siRNA (40 nM) were transfected into cells using Lipofectamine RNAiMax Reagent (Invitrogen, CA, USA) according to the manufacturer’s instructions. After 72 hours, transfected cells were subjected to subsequent analysis.

### Lentiviral EZH2 shRNA knockdown

Further evaluation of EZH2 knockdown in synovial sarcoma cells was carried out with lentiviral shRNA (Sigma-Aldrich, SHCLNV-NM_004456). The human EZH2 shRNA sequence targeted the coding region: 5′-CCGGTATGATGGTTAACGGTGATCACTCGAGTGATCACCGTTAACCATCATATTTTTG-3′. Briefly, synovial sarcoma cells were seeded (2  ×  10^3^ per well) in a 96-well microplate and incubated with EZH2 shRNA lentivirus for 24 hours at 37 °C in a humidified incubator in an atmosphere of 5% CO_2_. Untreated cells were used as blank control, and a lentiviral empty vector and lentivirus-based non-specific shRNA were used as negative controls. Each lentiviral construct and control was plated in duplicate wells. Hexadimethrine bromide was added to enhance transduction efficacy. Fresh medium containing 2 μg/ml puromycin, which is lethal to most lentivirus-untransduced cells, was exchanged every 3–4 days until resistant colonies were established. The knockdown effect of EZH2 shRNA was then evaluated by to cell proliferation analysis.

### Western blot

Protein lysates of the cells were extracted with 1×  RIPA lysis buffer (Upstate Biotechnology, Charlottesville, VA) supplemented with complete protease inhibitor cocktail tablets (Roche Applied Science, IN, USA). The protein concentrations were determined by Protein Assay Reagents (Bio-Rad, Hercules, USA). Equal amounts of protein were separated by NuPAGE^®^ 4–12% Bis-Tris Gel (Life Technologies), transferred onto nitrocellulose membrane (Bio-Rad), and incubated with specific primary antibodies EZH2 (Cell Signaling, Beverly, MA, dilution 1:1000), H3K27me3 (Active Motif, Carlsbad, CA, dilution 1:1000), and β-Actin (Sigma-Aldrich, St. Louis, MO, dilution 1:2000) at 4 °C overnight. The membranes were further probed with respective secondary antibodies (LI-COR Biosciences, Lincoln, NE), and scanned by Odyssey^®^ CLx equipment (LI-COR Biosciences) to detect the bands and the density.

### Wound healing assay

Cell migration activity was evaluated by wound healing assay. In brief, 2 × 10^5^ cells were seeded onto 12-well plates, and treatments were administered. After cells reaching 100% confluence, the cells were wounded by scraping of three parallel lines with a 200 μl tip, and then washed 3 times in serum-free medium and incubated in regular medium. Wounds were observed at 0, 8, 24, and 48 h. Three images were taken per well at each time point using a 10× objective, and the distance between the two edges of the scratch (wound width) was measured at three sites in each image. The cell migration distance was calculated by subtracting the wound width at each time point from the wound width at the 0 h time point.

### MTT cell proliferation assay and drug cytotoxicity assay

Synovial sarcoma cells were seeded at 1 × 10^4^ cells per well into 96-well plates, and treatments were administered. After the indicated time periods of incubation, 20 μL of MTT (5 mg/mL, dissolved in PBS, Sigma-Aldrich) was added to each well and the cells were cultured for 4 hours at 37 °C. The MTT formazan product was dissolved with acid isopropanol. The absorbance at a wavelength of 490 nm (A490) was measured on a SPECTRAmax Microplate Spectrophotometer from Molecular Devices (Sunnyvale, CA).

### Tissue Microarray Immunohistochemistry

The expression level of EZH2 was determined following the immunohistochemistry protocol (Paraffin) from Cell Signaling Technology (Beverly, MA) as previously described. The synovial sarcoma TMA was purchased from US Biomax, Inc. (Rockville, MD) containing 50 cases/100 cores with histopathologic data, including age, sex, tumor tissue type and location, clinical stage, TNM grading, and pathology grade. The information of “clinical stages” about this synovial sarcoma TMA is available at the vendor’s website (http://www.biomax.us/tissue-arrays/Soft_Tissue/SS1001). A Hematoxylin and Eosin stained tissue array slide was made available online by the manufacturer. Briefly, 5-μm paraffin tissue section slides of the TMA were baked for 1 hour at 60 °C, deparaffinized in xylene three times for 10 minutes each, and then transferred through graded ethanol (100% and 95%) twice for rehydration, 10 minutes each. After heat-induced epitope retrieval, the endogenous peroxidase was quenched by 3% hydrogen peroxide. Following protein blocking by normal goat serum for 1 hour at room temperature, the slide was incubated with the EZH2 primary antibody (dilution 1:50) at 4 °C overnight in a humidified chamber. Subsequently, bound antibody on the array was detected by SignalStain^®^ Boost Detection Reagent (Cell Signaling Technology) and SignalStain^®^ DAB (Cell Signaling Technology). The nuclei of synovial sarcoma cells were counterstained with hematoxylin QS (Vector Laboratories) to obtain better images. Finally, the section was mounted with VectaMount AQ (Vector Laboratories) for long-term preservation. EZH2 expression was evaluated semi-quantitatively according to nuclear staining. Staining patterns were categorized on a semi-quantitative scale from 0–5+ as follows: 0, no nuclear staining; A, 1+, <10% of cells stained positive; B, 2+, 10% to 25% positive cells; C, 3+, 26% to 50% positive cells; D, 4+, 51% to 75% positive cells; and E, 5+, >75% positive cells.

### Statistical analysis

The data were analyzed using Prism 5.0 software (Graph Pad Software Inc., San Diego, CA), and expressed as mean ± SEM. Statistical significance was assessed using independent two-tailed Student t-tests for independent data. Differences of *p *< 0.05 were considered significant for all statistical tests.

## Additional Information

**How to cite this article**: Shen, J. K. *et al.* Targeting EZH2-mediated methylation of H3K27 inhibits proliferation and migration of Synovial Sarcoma *in vitro. Sci. Rep.* **6**, 25239; doi: 10.1038/srep25239 (2016).

## Supplementary Material

Supplementary Information
