# Supplementary material for: Targeting EZH2-mediated methylation of H3K27 inhibits proliferation and migration of Synovial Sarcoma in vitro
Source: Sci Rep. 2016 Apr 29;6:25239. doi: 10.1038/srep25239 (PMC4850444; doi:10.1038/srep25239)
Supplement: Supplementary Information [file srep25239-s1.pdf]

Supplementary Information for:

Targeting EZH2-mediated methylation of H3K27 inhibits proliferation and migration of Synovial Sarcoma *in vitro*

Jacson K. Shen[1,+], Gregory M. Cote[2,+], Yan Gao[1], Edwin Choy[2], Henry J. Mankin[1], Francis J. Hornicek[1], Zhenfeng Duan[1,*]

[1]Sarcoma Biology Laboratory, Center for Sarcoma and Connective Tissue Oncology, Massachusetts General Hospital, Boston, United States

[2]Division of Hematology and Oncology, Massachusetts General Hospital, Boston, United States

[+] These authors contributed equally to this work

[*]Corresponding Author: Zhenfeng Duan, Sarcoma Biology Laboratory, Center for Sarcoma and Connective Tissue Oncology, Massachusetts General Hospital, 100 Blossom Street, Jackson 1115, Boston, MA 02114; Phone: 617-724-3144; Fax: 617-726-3883; E-mail: zduan@mgh.harvard.edu

Supplementary Figure S1. Correlation of EZH2 expression and histopathology of synovial sarcoma patient tissues. **a**, Representative images of nuclear staining intensity of EZH2 or HE staining in synovial sarcoma tumor tissues. **b**, Distribution of EZH2 staining scores between clinical stages.

Supplementary Figure S2. Determination of the fusion gene variant in the Aska-SS, Yamato-SS, Fuji, and SYO-1 synovial sarcoma cell lines by quantitative real time RT-PCR.

Supplementary Figure S3. The chordoma UCH2 cell line and the liposarcoma SW872 cell line treated with EPZ005687 over 14 days as evaluated by the MTT assay. They do not contain the SS18-SSX translocation, and thus, were used as controls.

Supplementary Figure S4. Migration images of EPZ005687 treated synovial sarcoma cell lines. SYO-1 and Aska-SS cells were treated with increasing concentrations of EPZ005687, and cell migration distances were measured over 48 hours.

Supplementary Figure S5. EPZ005687 in combination with etoposide, topotecan, or doxorubicin chemotherapy in SYO-1 and Aska-SS synovial sarcoma cell lines.

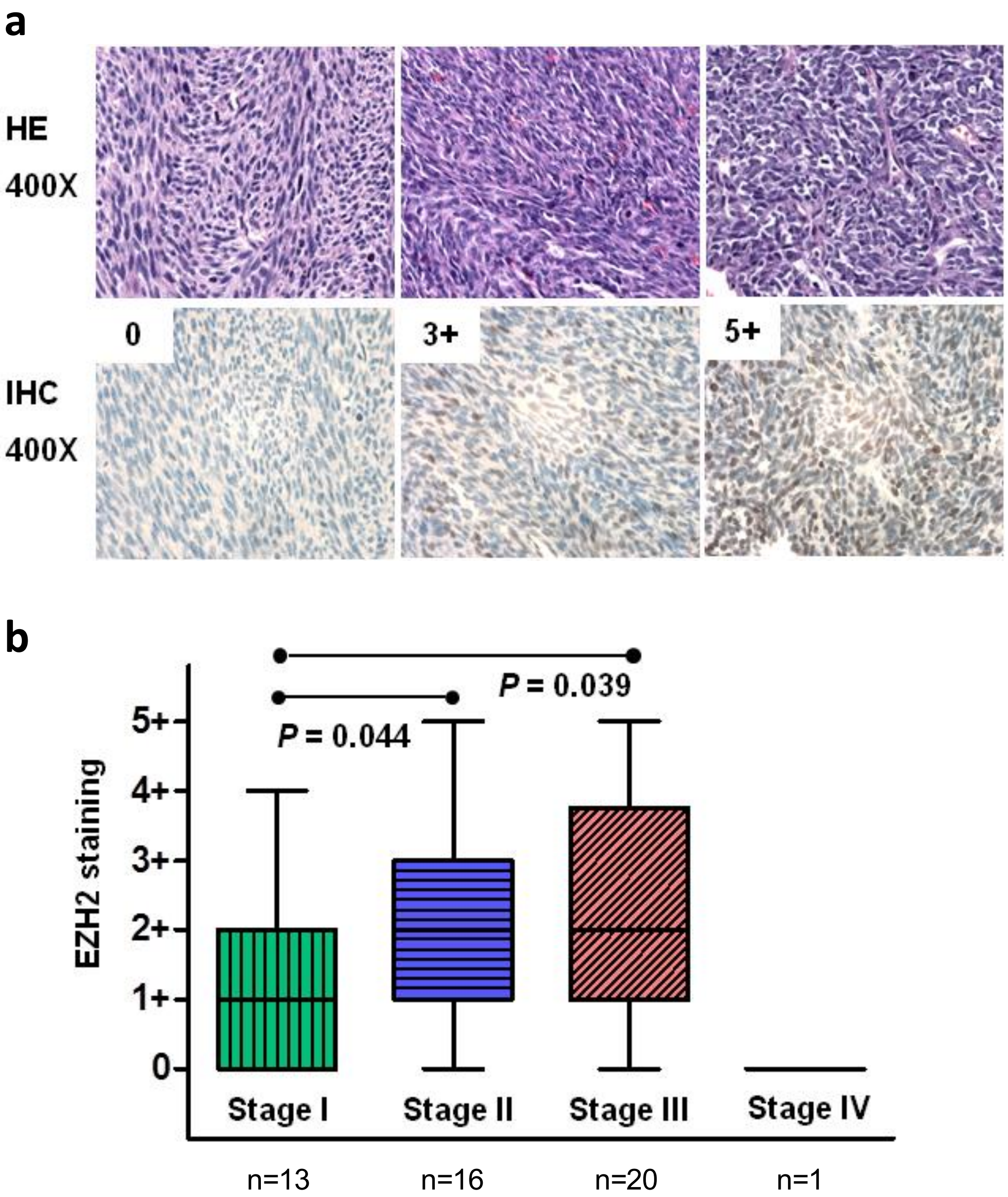
a
HE
400X
IHC
400X
0
3+
5+
b
P = 0.039
P = 0.044
EZH2 staining
5+
4+
3+
2+
1+
0
Stage I
Stage II
Stage III
Stage IV
n=13
n=16
n=20
n=1

Supplementary Figure S2

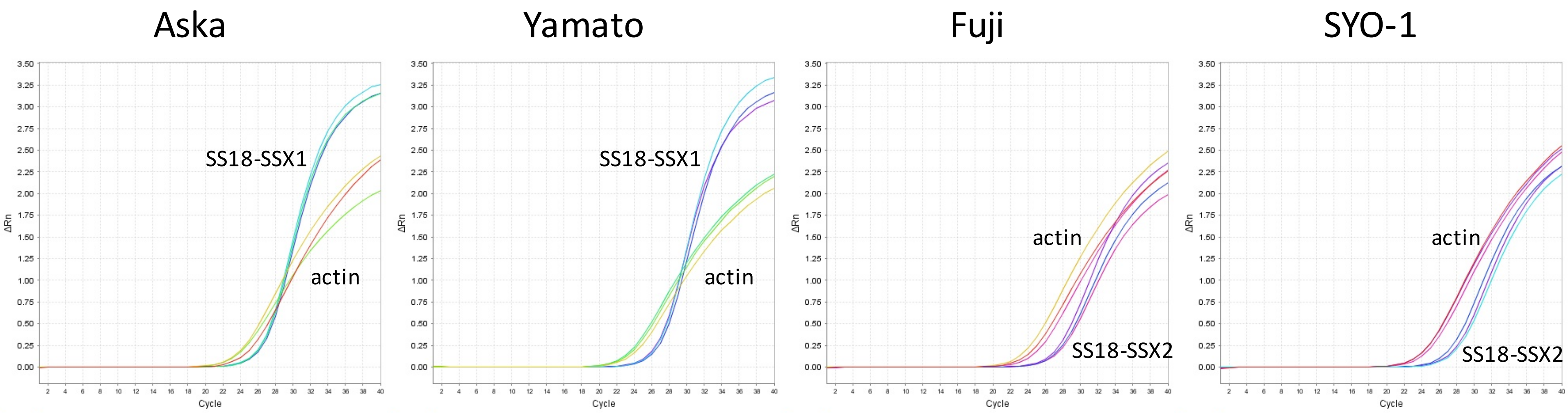

Cell line	Fusion protein variant
Aska	SS18-SSX1
Yamato	SS18-SSX1
Fuji	SS18-SSX2
SYO-1	SS18-SSX2

Supplementary Figure S3

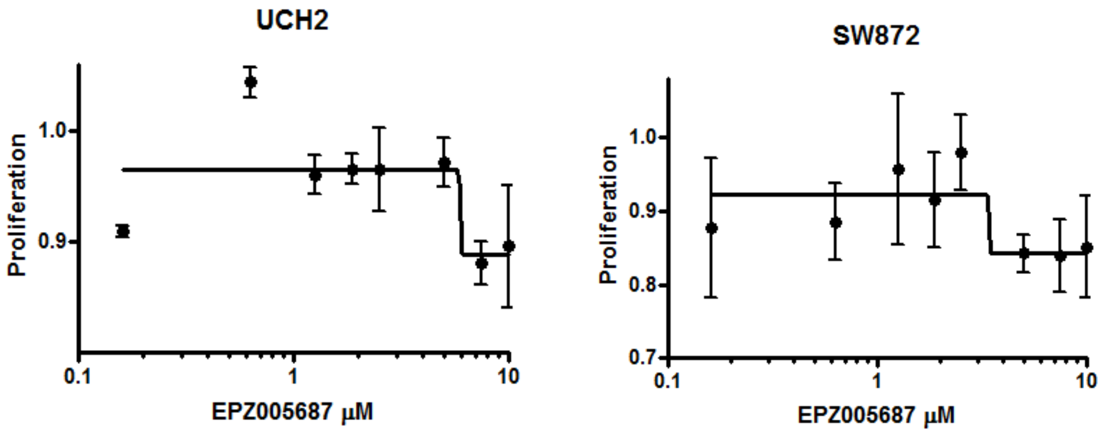

Supplementary Figure S4

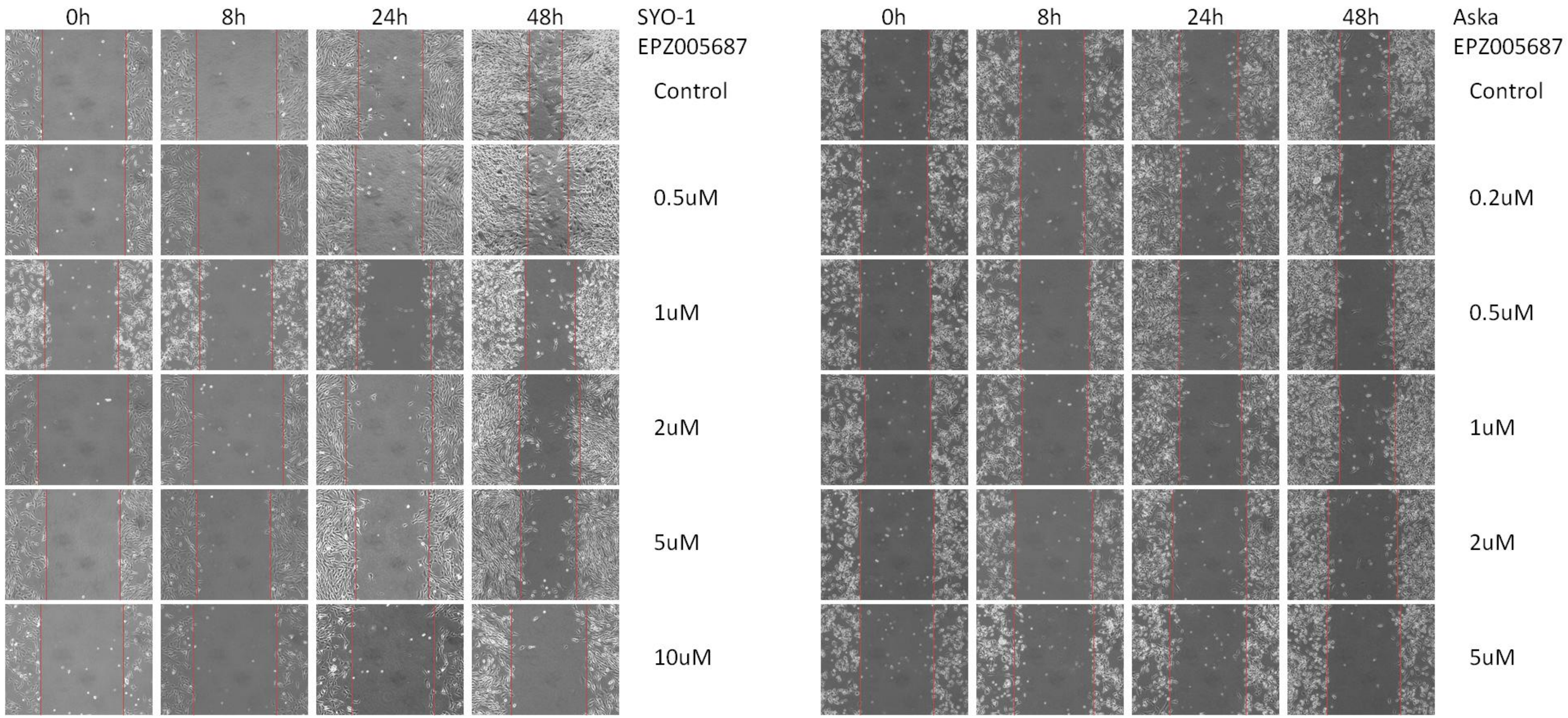

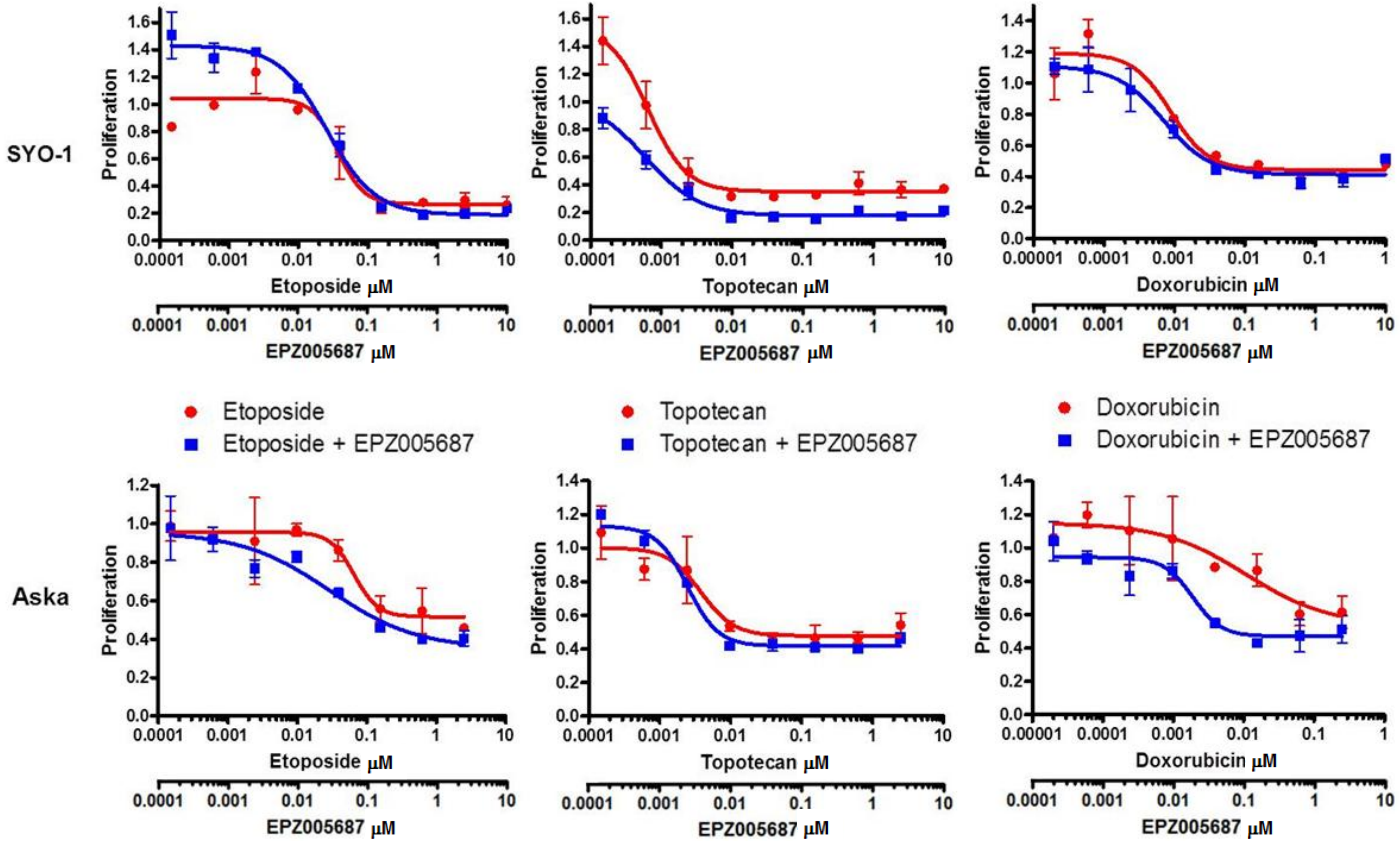
SYO-1
Aska
Proliferation
Etoposide μM
Topotecan μM
Doxorubicin μM
EPZ005687 μM
Etoposide
Etoposide + EPZ005687
Topotecan
Topotecan + EPZ005687
Doxorubicin
Doxorubicin + EPZ005687
